# Functional connectivity changes are correlated with sleep improvement in chronic insomnia patients after rTMS treatment

**DOI:** 10.3389/fnins.2023.1135995

**Published:** 2023-04-17

**Authors:** Lin Zhu, Ge Dang, Wei Wu, Junhong Zhou, Xue Shi, Xiaolin Su, Huixia Ren, Zian Pei, Xiaoyong Lan, Chongyuan Lian, Peng Xie, Yi Guo

**Affiliations:** ^1^Department of Neurology, Shenzhen People’s Hospital, The Second Clinical Medical College, Jinan University, The First Affiliated Hospital, Southern University of Science and Technology, Shenzhen, Guangdong, China; ^2^Department of Psychiatry and Behavioral Sciences, Stanford University, Stanford, CA, United States; ^3^Hebrew Seniorlife, Hinda and Arthur Marcus Institute for Aging Research, Harvard Medical School, Boston, MA, United States; ^4^Department of Geriatrics, Shenzhen People’s Hospital, The Second Clinical Medical College, Jinan University, The First Affiliated Hospital, Southern University of Science and Technology, Shenzhen, Guangdong, China; ^5^Shenzhen Bay Laboratory, Shenzhen, Guangdong, China; ^6^NHC Key Laboratory of Diagnosis and Treatment on Brain Functional Diseases, The First Affiliated Hospital of Chongqing Medical University, Chongqing, China

**Keywords:** chronic insomnia disorder, rTMS, EEG, functional connectivity, DLPFC

## Abstract

**Background:**

Repetitive transcranial magnetic stimulation (rTMS) has been increasingly used as a treatment modality for chronic insomnia disorder (CID). However, our understanding of the mechanisms underlying the efficacy of rTMS is limited.

**Objective:**

This study aimed to investigate rTMS-induced alterations in resting-state functional connectivity and to find potential connectivity biomarkers for predicting and tracking clinical outcomes after rTMS.

**Methods:**

Thirty-seven patients with CID received a 10-session low frequency rTMS treatment applied to the right dorsolateral prefrontal cortex. Before and after treatment, the patients underwent resting-state electroencephalography recordings and a sleep quality assessment using the Pittsburgh Sleep Quality Index (PSQI).

**Results:**

After treatment, rTMS significantly increased the connectivity of 34 connectomes in the lower alpha frequency band (8–10 Hz). Additionally, alterations in functional connectivity between the left insula and the left inferior eye junction, as well as between the left insula and medial prefrontal cortex, were associated with a decrease in PSQI score. Further, the correlation between the functional connectivity and PSQI persisted 1 month after the completion of rTMS as evidenced by subsequent electroencephalography (EEG) recordings and the PSQI assessment.

**Conclusion:**

Based on these results, we established a link between alterations in functional connectivity and clinical outcomes of rTMS, which suggested that EEG-derived functional connectivity changes were associated with clinical improvement of rTMS in treating CID. These findings provide preliminary evidence that rTMS may improve insomnia symptoms by modifying functional connectivity, which can be used to inform prospective clinical trials and potentially for treatment optimization.

## Introduction

Insomnia is the most prevalent sleep disorder and is associated with difficulties in initiating or maintaining sleep, as well as a decline in daytime performance and cognitive impairment, leading to many health issues (Spiegelhalder et al., 2015). Hypnotics are often prescribed as a first-line treatment for acute insomnia (Glass et al., 2005); however, the long-term use of hypnotics has adverse effects and may confer a risk of dependence. Cognitive behavioral therapy (CBT) is an alternative to chronic pharmacological insomnia treatment. Although there is evidence for long-lasting improvement in sleep after CBT, the strict time commitment and insufficient number of qualified clinicians to employ it, limit its use in clinical practice (Morin and Benca, 2012). Therefore, better treatment options for chronic insomnia, a long-term pattern of difficulty sleep, are urgently needed.

Insomnia is rarely an isolated psychobiological disorder and is associated with measurable aberration in functional brain mechanisms (Riemann et al., 2010). Neuroimaging studies, such as electroencephalography (EEG) or functional magnetic resonance imaging (fMRI), showed that patients with insomnia have abnormal connectivity patterns in emotional circuits (Huang et al., 2012), salience networks (Chen et al., 2014), and brain network topology in general (Li et al., 2018). Moreover, previous studies have suggested that increased cortical excitability was associated with chronic insomnia (Lanza et al., 2015; Ly et al., 2016).

Repetitive transcranial magnetic stimulation (rTMS) can noninvasively modulate cortical activity by delivering a sequence of magnetic pulses. Normally, low frequency (<1 Hz) is thought to inhibit, and high frequency (≥5 Hz) to facilitate motor cortical excitability (Fitzgerald et al., 2006). The inhibitory effect of low frequency (i.e., 1 Hz) rTMS on cortical excitability has therefore led to it being increasingly considered for the treatment of insomnia disorders.

Stimulation site is another essential factor in the field of clinical application of rTMS. Dorsolateral prefrontal cortex (DLPFC) is the most widely used rTMS target for the treatment of neuropsychiatric disorders, including depression (Eshel et al., 2020). As a node of the frontoparietal network, it plays a critical role in integrating cognition and emotion (Mars and Grol, 2007; Gong et al., 2020). According to a previous neuroimaging study, the DLPFC of insomnia patients shows hyperexcitability compared with those who were well-slept (Spiegelhalder et al., 2013). As a result, low frequency DLPFC rTMS appears to be a reasonable strategy for the treatment of insomnia (Gong et al., 2020).

The efficacy of rTMS in treating insomnia has been investigated in several clinical studies. Most of them chose the DLPFC as the target. These studies found that 10 daily sessions of low frequency rTMS stimulation applied to the right or bilateral DLPFC resulted in a significant decrease in the Pittsburgh Sleep Quality Index (PSQI; lower index indicates better sleep) (Jiang et al., 2013; Feng et al., 2019; Shi et al., 2021). Only one study selected the right posterior parietal cortex as the stimulation site and reported similar results: 14 consecutive low frequency rTMS sessions could lower down PSQI and Insomnia Severity Index significantly (Song et al., 2019).

However, despite the aforementioned clinical evidences, there are limited neurophysiological studies investigating the underlying mechanisms of low frequency DLPFC rTMS in patients with chronic insomnia. In this context, we conducted this single-arm, open-label, interventional study to determine the neural mechanisms of rTMS other than to demonstrate its clinical efficacy. We used the resting-state electroencephalography (rsEEG) to assess the functional connectivity characteristics in patients with chronic insomnia disorder (CID) before, after and 1-month after 10 daily rTMS sessions. rsEEG is a promising paradigm for studying abnormal functional architectures in various disorders due to its task-independent properties (Zhang et al., 2021). The overarching goal was to investigate the alterations of functional connectivity induced by rTMS in patients with CID, which is critical for linking translationally relevant discoveries that can be applied in a clinical setting. The secondary aim of this study was to examine whether the clinical improvement observed 1 month after rTMS was related to any previously identified connectivity characteristics. We hypothesized that rTMS would induce connectivity changes and that such changes would correlate significantly with symptom improvements.

## Materials and methods

### Participants

Participants with CID were recruited at the Neurology Department of Shenzhen People’s Hospital. A total of 47 patients were screened for eligibility, of whom 37 gave informed consent to participate in this study. All experimental details were approved by the Ethics Committee of Shenzhen People’s Hospital (see chictr.org.cn registration: ChiCTR1900026904). Nine patients dropped out during the 1-month follow-up period; therefore, only 28 patients were included in the statistical analysis of follow-up.

All patients were required to meet the diagnostic criteria for CID according to the International Classification of Sleep Disorders, Third Edition (ICSD-3). The inclusion criteria were as follows: (1) aged 18–70 years, right-handed; (2) the sleep disturbances occur at least three times per week and present for the last 3 months; (3) PSQI ≥7 (Buysse et al., 1989); (4) scored <25 on 24-item Hamilton Depression Rating Scale (HAMD); (5) no other sleep disorders like sleep apnea, etc.; and (6) failure of at least one adequate sleep medication trial. The exclusion criteria were: (1) any contraindication to TMS (history of seizures, metallic implants, etc.); and (2) prior history of neurological or psychiatric disorders.

In addition, 40 healthy controls (HC) without sleep problems participated in the baseline assessment, serving as a reference for changes in functional connectivity. HCs needed to meet the following criteria: (1) no history of sleep disorders; (2) PSQI <7; (3) HAMD ≤7 and Hamilton Anxiety Rating Scale (HAMA) ≤7 (Matza et al., 2010; Zimmerman et al., 2013); and (4) no neurological or psychiatric disorders.

During the study, patients were allowed to take concomitant medications, and were asked to remain constant throughout the clinical trial (see Supplementary Methods for details).

### rTMS treatment

Stimulation was performed using a figure 8-shaped focal coil attached to a MagPro 100 magnetic stimulator (MagVenture, Copenhagen, Denmark). All patients received 1 Hz (10 s trains, 1 s inter-train interval, 1,360 pulses per session) rTMS treatment once daily on weekdays for 2 consecutive weeks. rTMS was delivered over the right DLPFC (F4 electrode site according to the International 10–20 EEG system) at 100% of the resting motor threshold (RMT) (Mir-Moghtadaei et al., 2015). To determine the RMT, stimulus intensity was gradually increased until 5 out of 10 trials elicited motor evoked potentials with peak-to-peak amplitudes over 50 μV in the contralateral abductor pollicis brevis muscle (Rossini et al., 2015). Adverse events attributed to rTMS were documented and reported.

### Clinical assessment

**Figure 1:**
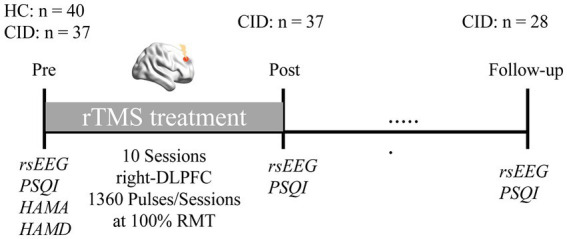
Experimental protocol. Healthy controls (HC) and patients with chronic insomnia disorder (CID) participated in an EEG session before rTMS treatment, as well as clinical assessments. Afterwards, patients received a course of low frequency rTMS treatment targeting the right DLPFC. Subsequently, EEG data and PSQI were collected again after treatment, and at 1-month follow-up, respectively.

All participants received a pre-treatment assessment with the PSQI, HAMA, and HAMD. We used the PSQI to measure sleep quality, as well as HAMA and HAMD to assess participant’s anxious and depressive states. Likewise, the PSQI was measured post-treatment (upon completion of the final treatment session) and at 1-month follow-up. In order to determine the magnitude of the rTMS response, the percent reduction in PSQI from pre- to post-treatment, and from pre-treatment to follow-up, were calculated (see Figure 1 for the experimental design).

### Electroencephalography

For the HC group, EEG data were recorded once at baseline. For the CID patients, EEG data were acquired at three time points concurrently with PSQI. Equipment setting and environment were uniform across all three acquisitions.

EEG recordings were acquired from 64 channels under closed-eye conditions for 8 min using a BrainAmp DC amplifier (Brain Products GmbH, Germany). During the recording, the participants were asked to relax on a comfortable chair in a metal-shielded room. The data were referenced online to the FCz channel with the ground at AFz. Data were initially sampled at 5,000 Hz with impedances kept below 5KΩ throughout the data collection period. Participants were instructed to refrain from consuming any caffeinated (or energy) drinks within 24 h of the EEG recording sessions.

The details of the pre-processing and source localization are described in the Supplementary Methods. Based on previous findings, the resulting EEG data were filtered into five frequency bands: theta (4–8 Hz), low-alpha (8–10 Hz), high-alpha (10–13 Hz), beta (13–30 Hz), and gamma (30–45 Hz).

### Estimating functional connectivity

All connectivity analyses were computed at the source level using 3,003 vertices and then projected into 31 cortical regions of interest (ROI) using the Montreal Neurological Institute template (Chen et al., 2013); (see Supplementary Table 1). Here, we chose the debiased weighted phase-lag index (dwPLI) to represent the non-zero phase-lag statistical interdependencies between each pair of ROIs (Vinck et al., 2011). dwPLI is an optimized phase lag index that minimizes the influence of volume conduction and field spread, which could affect the estimation even at the source level. The connectivity between each pair of regions was calculated by averaging the dwPLI values over all possible vertex pairs. Accordingly, we identified 465 edges representing each participant’s regional pairwise connectivity.

### Statistical analysis

To compare the difference in clinical outcomes, we analyzed the PSQI of pre-, post-treatment and follow-up using a linear mixed model with a fixed effect of time and a random intercept.

The differences between pre- and post-functional connectivity matrices were analyzed using the Network-based Statistics (NBS) (Zalesky et al., 2010), a nonparametric statistical test to control for the family-wise error rate resulting from multiple comparisons. For each comparison, 5,000 random permutations were used.

To further investigate the association of functional connectivity changes with clinical outcomes, a multiple linear regression model was constructed between the pre- to post-network differences and the percentage PSQI change with age, sex, HAMA, and HAMD as covariates. Here, all edges that varied significantly (NBS-corrected, *p* < 0.01) between the pre- and post- functional connectivity matrices were treated as independent inputs. When implementing feature selection, we used the least absolute shrinkage and selection operator (LASSO) to define a low-dimensional representation of the selected connectivity features (Tibshirani, 1996). Correlation analysis between the estimated and the actual PSQI value of post-treatment and follow-up were performed using Pearson’s correlation.

## Results

### Clinical results

The demographic and clinical characteristics of the two groups are summarized in Table 1. There were significant differences in the PSQI, HAMA, and HAMD scores (*p* < 0.05) between the HC and CID groups. No significant differences were found in age or sex. Among these characteristics, the PSQI global score correlated appreciably with HAMD score (*p* < 0.01, *r* = 0.447) but was not associated with the other measures.

**Table 1:** Demographic and clinical characteristics of the participants.

Variables	HC (*n* = 40)	CID (*n* = 37)	*p*-value
Age, year	23–72, 46.1 ± 9.4	22–69, 48.9 ± 11.1	0.23
Sex (M/F)	18/22	15/22	0.69
PSQI	3.2 ± 1.6	15.1 ± 3.4	<0.001
HAMA	2.6 ± 1.8	13.6 ± 6.2	<0.001
HAMD	2.7 ± 1.8	13.3 ± 5.0	<0.001
RMT	/	39.0 ± 12.5	

Data are presented as mean ± SD. The *p* values were obtained by independent sample *t*-test and chi-square test (for sex only). HC, healthy control; CID, chronic insomnia disorder; PSQI, Pittsburgh Sleep Quality Index; HAMA, Hamilton Anxiety Rating Scale; HAMD, Hamilton Depression Rating Scale; RMT, resting motor threshold.

All patients tolerated the rTMS well, and no adverse effects were reported. A mixed-effects model assessing PSQI differences revealed a significant main effect of rTMS treatment [*F*(1.84, 57.88) = 12.38, *p* < 0.0001]. *Post hoc* comparisons indicated that rTMS facilitated a significant reduction in insomnia symptoms on the PSQI between pre- and post-treatment (delta-PSQI = 3.243, *p* = 0.0002, Cohen’s *d* = 0.857), and between scores pre- and 1-month later (delta-PSQI = 3.911, *p* = 0.0003, Cohen’s *d* = 1.132) (see the Supplementary Figure 1).

### rTMS-induced functional connectivity changes and association to clinical outcomes

**Figure 2:**
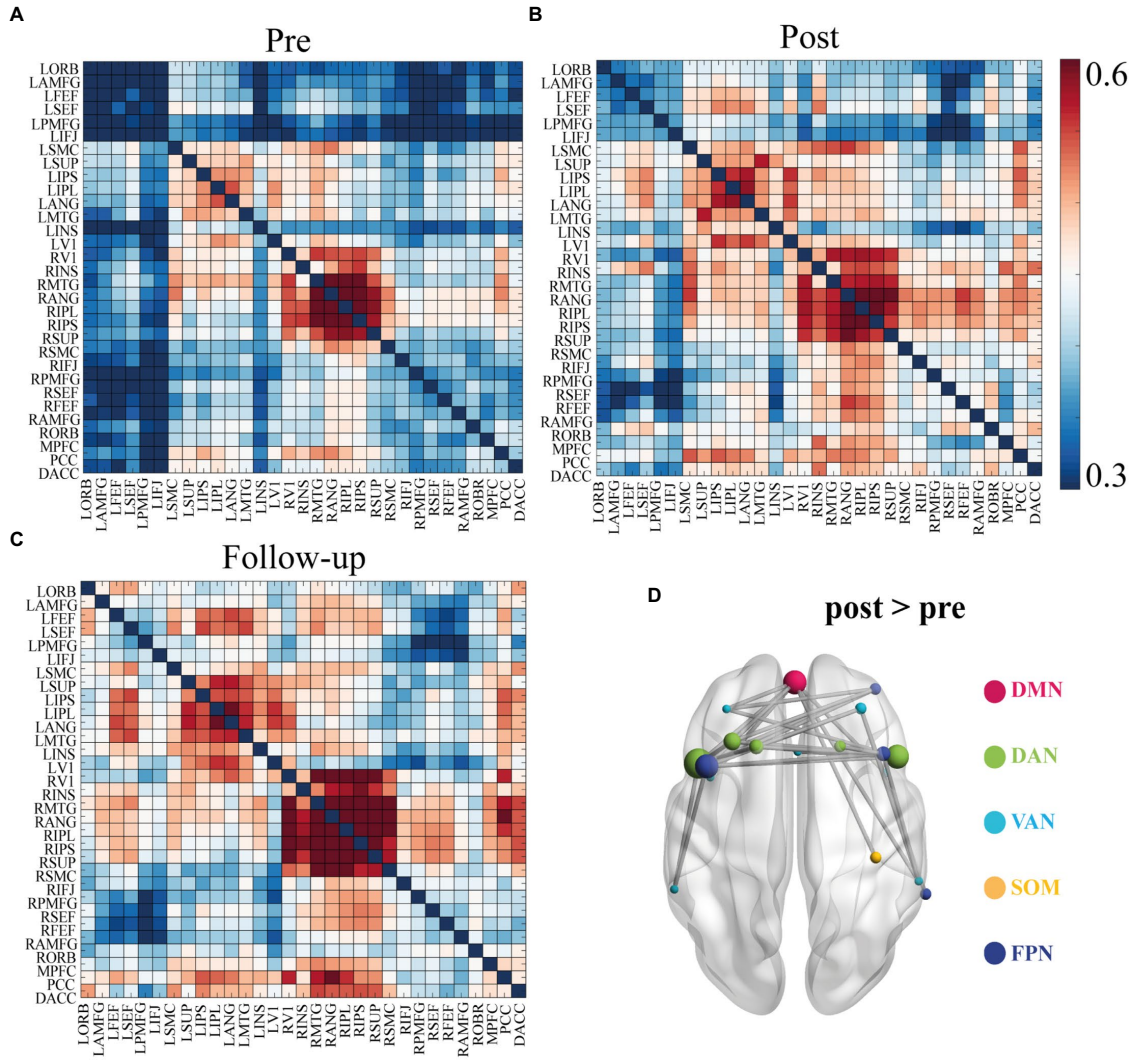
Functional connectivity matrices in low alpha band (8–10 Hz) and 34 identified edges. **(A)** Connectivity matrices of pre-treatment EEG scan. **(B)** Connectivity matrices of post-treatment EEG scan. **(C)** Connectivity matrices of the follow-up EEG scan. **(D)** Significant functional connectivity changes between the pre- and post- EEG scans included 34 edges. Significance was determined by network-based statistics (NBS) correction at *p* < 0.01. List of the brain regions are presented in the Supplementary Table 1. List of the 34 pairs showed significant differences are presented in the Supplementary Table 3. DMN, default mode network; DAN, Dorsal attention network; VAN, Ventral attention network; SOM, somatosensory network; FPN, frontoparietal network.

There were no significant differences in the functional connectivity matrices between the pre- and post-treatment measures that survived multiple corrections at four frequency bands: theta (4–8 Hz), high-alpha (10–13 Hz), beta (13–30 Hz), and gamma (30–45 Hz). Instead, a comparison of the functional connectivity of low-alpha frequency band (8–10 Hz) identified 34 edges that had been significantly changed at *p* < 0.01 level after NBS correction (Figure 2; also see Supplementary Table 2). Specifically, CID patients had a lower mean functional connectivity pre-treatment. On average, these rTMS-induced connectivity changes brought patients’ patterns aligned more closely with healthy controls. At follow-up, the strength of the connectivity matrix remained relatively stable (see Figure 2C). The most frequently occurring connectomes were located in the frontal lobe, preferentially involving the frontoparietal network (FPN), dorsal attention network (DAN), and ventral attention network (VAN) (see Figure 2D). Moreover, a dominant interhemispheric functional connectivity change was also notable.

**Figure 3:**
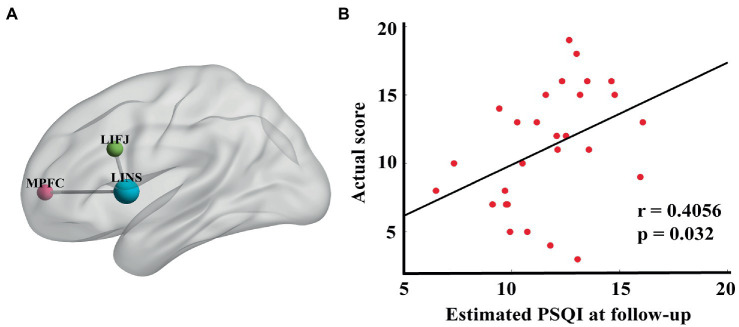
**(A)** Edges that were significantly correlated with percentage PSQI change before and after treatment (LIFJ-LINS, *p* < 0.01, MPFC-LINS, *p* < 0.01). **(B)** The estimated PSQI at follow-up were significantly correlated with the actual score (*r* = 0.4056, *p* = 0.032), with age, gender, HAMA and HAMD at baseline as the nuisance covariates.

Regression analyses were performed to identify connections that were significantly correlated with improvement in PSQI scores at the end of rTMS treatment. That is, the functional connectivity changes between pre- and post-treatment of these 34 identified edges were taken as independent variables (see Supplementary Table 3 for coefficients of regression model). Notably, two edges appeared to be significantly correlated with PSQI change (*r* = 0.62, *p* = 0.02) (see Figure 3B): the left inferior frontal junction to left insula (LIFJ-LINS) and the medial prefrontal cortex to left insula (MPFC-LINS) (see Figure 3A).

### Analysis of connectivity changes and clinical outcomes at follow-up

EEG data acquired 1 month after treatment were used to validate the association between the connectomes obtained in the previous regression model and clinical measures. The connectivity changes of these two edges between the pre- and follow-up assessments were taken as independent inputs, including age, sex, HAMA, and HAMD at baseline, as covariates. The estimated PSQI score was significantly correlated with the actual PSQI score collected 1 month after rTMS completion (*r* = 0.41, *p* = 0.032) (Figure 3B).

### Longitudinal connectivity analysis

**Figure 4:**
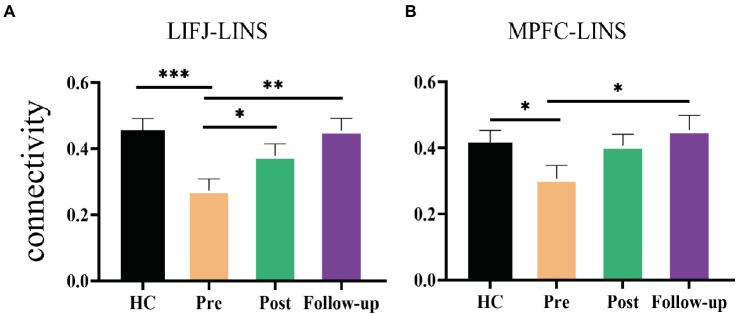
Significant rTMS effect of group and time. **(A)** Changes of the mean functional connectivity between left inferior eye junction (LIFJ) and left insula (LINS). Mixed effect model revealed significant rTMS treatment effect in the functional connection [*F*(1.8, 55.83) = 7.256, *p* = 0.0022]. *Post hoc* comparison showed significant differences between pre- and post-treatment (*p* = 0.0236), and pre-treatment to follow-up (*p* = 0.0076). The functional connectivity of HC was significantly higher than CID patients at baseline (*t* = 3.641, *p* = 0.0005). **(B)** Changes of the mean functional connectivity between medial prefrontal cortex (MPFC) and left insula (LINS). Mixed effect model revealed significant rTMS treatment effect in the functional connection [*F*(1.92, 59.53) = 3.862, *p* = 0.028]. *Post hoc* comparison showed a significant difference between pre-treatment and follow-up (*p* = 0.0497). Independent *t*-test showed that CID patients had significantly lower functional connectivity at baseline than HCs (*t* = 2.016, *p* = 0.0474). Error-bars represents SEM. *p < 0.05, **p < 0.01, ***p < 0.001.

To gain further insight into the rTMS-induced changes in LIFJ-LINS and MPFC-LINS connectivity, we examined mean values at each time point for both HC and CID groups. Significant difference was observed between the HC and CID pre-treatment for both LIFJ-LINS (average 0.46 vs. 0.27) and MPFC-LINS (average 0.41 vs. 0.30) connections (see Figure 4). After rTMS, the connectivity of the LIFJ to the LINS increased significantly at post-treatment and follow-up. The trend of MPFC-LINS connectivity resembled a similar pattern, which increased significantly at follow-up compared to that at baseline. These active rTMS-induced changes shifted the CID patient profile closer to that of HCs (see pairwise statistics in the Figure 4 legend).

## Discussion

To better understand the regulatory role of rTMS in patients with CID, we examined longitudinal changes in functional connectivity induced by low frequency rTMS of the right DLPFC. In agreement with previous studies, our results confirmed the efficacy of this low frequency rTMS protocol for treating clinical CID. We found that rTMS to the right DLPFC was associated with widespread alterations in functional connectivity, which correlated with clinical outcomes, and this association persisted 1 month after the cessation of rTMS stimulation. These results offer promising preliminary evidence linking the altered brain function patterns observed in CID with clinical symptoms and expands on how changes in both aspects are associated.

Unlike other studies that focused only on *a priori* brain regions, we discovered connectivity biomarkers based directly on EEG data without making any assumptions. This data-driven approach allows us to account for uncertainties in the spatial distribution of connectivity changes correlated with clinical outcomes. Our findings suggest that the significant increase in connectivity is not confined to the ones within the stimulated network, but instead spreads across networks, involving the FPN, DAN, and VAN. Remote functional connections are impacted more than local ones, indicating that rTMS ultimately modulates insomnia-related connectivity (Castrillon et al., 2020). Additionally, we observed a dominant interhemispheric functional connectivity change, which is consistent with that reported in previous studies (Watanabe et al., 2014). Of note, since all the significant edges show higher connectivity after rTMS than before it, this observation corroborates that rTMS treatment may rewire impaired intracortical connections.

Neuroplasticity describes the ability of the brain to change and adapt its organization as a result of external influences. The results of the longitudinal analysis suggest that rTMS has a long-lasting plastic effect; hence, decrease in clinical symptoms and increase in functional connectivity characteristics could persist even 1-month after the completion of rTMS (Ge et al., 2020). It also proves the robustness of these two indicators, LINS-MPFC, LINS-LIFJ, over a long period of time.

The insula is a key node in the salience network, and its abnormalities have been pointed out in many studies of insomnia (Motomura et al., 2021). An fMRI research demonstrated decreased connectivity between the amygdala and insula during resting states, indicating a possible neural mechanism responsible for the sleep-related affective disorders and dysregulation of emotional control (Li et al., 2014). We found that patients with CID suffered significantly higher HAMA and HAMD than those with HCs, which reflects the true situation in real life. Our study reinforces earlier findings about the insula in CID and adds to the evidence that it plays a role in the disease’s pathophysiology. The MPFC and its ample connections with other regions, play critical roles in long-range, recent, and short-term memory over a wide range of activities (Euston et al., 2012). It has also been previously discussed regarding insomnia with the conclusion that circuit dysfunction involving the MPFC was associated with poor sleep quality as measured by the PSQI (Shao et al., 2020). The IFJ is part of the cognitive control network that co-activates with the DLPFC, ventrolateral prefrontal cortex, anterior insula, and posterior parietal cortex (Sundermann and Pfleiderer, 2012).

In light of our understanding of how alpha oscillations correlate with resting wakefulness, the therapeutic benefits of rTMS typically focus on alpha rhythms. The alpha rhythms promote coordination between cortical areas and between the cortex and subcortical structures, such as the thalamus (Buzsáki and Draguhn, 2004). A highly synchronized alpha rhythms can facilitate the coordination of brain regions in an event-related, synchronized readiness manner, prior to engaging in different type of tasks (Klimesch et al., 2007). We found that DLPFC-rTMS significantly enhanced the connectivity of low-frequency alpha rhythms (8–10 Hz), but not of the entire alpha band (8–13 Hz) (see Supplementary Figure 2). In the thalamo-cortical and cortico-cortical loops, alpha rhythms carry out different functions; for example, low-frequency alpha rhythms execute inhibitory functions (Pfurtscheller and Lopes da Silva, 1999). Researchers have proposed that the lower alpha band diffusely regulates alertness and arousal in the brain (Klimesch et al., 1998). Our results show that inhibitory 1 Hz rTMS stimulation of the DLPFC increases lower alpha synchronization in the resting state. This inhibitory synchronization state could facilitates recruitment of specific regions of the cortex to transmit and retrieve task information before preparing for subsequent tasks (Klimesch, 2012). A previous study reported a similar phenomenon. rTMS targeting in the angular gyrus, core regions of the DAN, enhanced intrahemispheric alpha coherence of 8–10 Hz, suggesting the causal role of the angular gyrus in modulating of dominant low-frequency alpha rhythms under the resting conditions (Capotosto et al., 2014).

CID appears to be associated with altered cortico-thalamic connectivity, which is partly responsible for cognitive regulation and circadian processes (Zou et al., 2021). Indeed, the thalamus is the pacemaker of cortical alpha rhythms. Taken together, we infer that DLPFC-rTMS regulates altered alpha connectivity by top-down control of DLPFC to the thalamus. While path analysis indicates a causal relationship, our study cannot infer causality since no connectivity with subcortical regions was observed. These results should therefore, only be considered as supportive. Further neuroimaging study is required to elucidate the potential involvement of the thalamus in the improvement of cortical alpha band connections following treatment. A previous study supports our hypothesis reporting that DLPFC-rTMS modulates the functional connectivity of the insula and thalamus in smokers (Li et al., 2017).

rTMS is a promising strategy for treating various neuropsychiatric disorders. The effects of rTMS depend on its intensity, frequency and stimulation site, in particular the stimulation site. However, there is no consensus on the optimal stimulation site for insomnia due to the prior lack of therapeutic mechanisms of rTMS. These findings offer implications for optimizing brain stimulation therapy, which should be examined in future clinical trials. Besides, as the optimal treatment parameters for insomnia have not yet been determined, the schedule implemented in this study is a commonly used protocol to ensure treatment adherence. It is essential to identify efficacious dosing parameters that are feasible for further studies.

This study has some limitations. First, the lack of a sham-control group prevents us from determining whether the results can be partly explained by placebo effects. In effect, the primary goal of this study was to determine the neural mechanism of rTMS rather than to demonstrate differential clinical outcome with active versus sham conditions. Second, chronic insomnia is quite a heterogenic disorder, often comorbid with other symptoms. To ensure the homogeneity of patients enrolled in the study, we strictly enrolled patients who had difficulties with sleep for over 3 months and had failed at least one medication trial. Another potential weakness of this study was that all patients were medicated. The drug itself affects EEG parameters. Ideally, the inclusion of medication-free patients would eliminate any potential confounding effect of medication; this is not an ethically viable option for patients with medication-resistant insomnia. It remains uncertain whether the functional connectomes found in the present study are general biomarkers of response across all treatments. Hence, these findings need to be replicated in equally large samples obtained from multiple sites.

## Conclusion

Despite its high prevalence, there has been little progress in the treatment of CID. We found that low frequency rTMS treatment over the right DLPFC significantly increased the EEG-derived functional connectivity in patients with CID. Furthermore, changes in connections, particularly the LIFJ-LINS and MPFC-LINS, were significantly associated with clinical measures and could predict the PSQI in the subsequent assessment at 1-month follow-up. These results could be very valuable in clinical treatment of insomnia, especially in patients that are resistant to medications or have a history of drug failure. These findings expand our understanding of neural response in patients with chronic insomnia treated with rTMS and lay the foundation for future studies. Further research using a rigorous design is required to address the aforementioned issues.

## Data availability statement

The raw data supporting the conclusions of this article will be made available by the authors, without undue reservation.

## Ethics statement

The studies involving human participants were reviewed and approved by the Shenzhen People’s Hospital. The patients/participants provided their written informed consent to participate in this study.

## Author contributions

LZ contributed to the data analysis and original draft. GD, XSh, and XSu were responsible for acquisition and analysis. WW and JZ assisted with analysis and manuscript revision. XL and CL contributed to the data collections. ZP and HR contributed to the data curation. PX provided clinical study consultation. YG was responsible for the conceptualization and funding acquisition. All authors critically reviewed the final content and approved for publication.

## Funding

This study was supported by the Natural Science Foundation of Guangdong Province (grant number 2021A1515010983), the Sanming Project of Medicine in Shenzhen (grant number SZSM202111009), the Shenzhen Science and Technology Innovation Program (grant number KCXFZ20201221173400001), and Shenzhen Key Medical Discipline Construction Fund (grant number SZXK005).

## Conflict of interest

The authors declare that the research was conducted in the absence of any commercial or financial relationships that could be construed as a potential conflict of interest.

## Publisher’s note

All claims expressed in this article are solely those of the authors and do not necessarily represent those of their affiliated organizations, or those of the publisher, the editors and the reviewers. Any product that may be evaluated in this article, or claim that may be made by its manufacturer, is not guaranteed or endorsed by the publisher.
