# Supplementary material for: Functional connectivity changes are correlated with sleep improvement in chronic insomnia patients after rTMS treatment
Source: Front Neurosci. 2023 Apr 17;17:1135995. doi: 10.3389/fnins.2023.1135995 (PMC10149758; doi:10.3389/fnins.2023.1135995)
Supplement: Supplementary file 1 [file Data_Sheet_1.docx]

Supplementary Material

Functional Connectivity Changes are Correlated with Sleep Improvement in Chronic Insomnia Patients after rTMS Treatment

# Supplementary Method

# *Participants*

Initially, 47 patients with chronic insomnia disorder (CID) were assessed for eligibility. 3 patients were excluded due to low PSQI, 3 patients were excluded due to high HAMD and 4 patients withdrew before the primary endpoint. Finally, 37 patients consented to participate in this study. Of these, 28 patients completed the entire study, 9 dropped out during the 1-month follow-up. Meanwhile, to ensure the homogeneity of patients enrolled in the study, all CID patients had failed at least one adequate sleep medication trial before. During the study, patients were allowed to take concomitant medications, and were asked to remain constant throughout the clinical trial. Here we included the details of medication usage: sleep aids (N = 28), including benzodiazepines and non-benzodiazepines; antidepressants (N = 23); and antipsychotics (N = 2).

Depressive symptoms were assessed in patients using the Hamilton Depression Rating Scale (HAMD). Patients were allowed to have comorbid anxiety symptoms but no depression, and were excluded if they met diagnostic criteria for any psychiatric disorders. In fact, the majority of the CID patients also scored higher on Hamilton Depression Rating Scale HAMD than healthy controls, which is difficult to avoid in practice.

Pittsburgh sleep quality index (PSQI) is a self-rated questionnaire that distinguishes good and poor sleepers. The PSQI global score has a range of 0-21 points. The Hamilton depression rating scale (HAMD) is the most popular clinician-administered depression assessment scale. The 24-item HAMD ranges from 0-76. Higher HAMD level indicate more severe depressive symptoms. Hamilton anxiety rating scale (HAMA) was developed to measure the severity of anxiety symptoms, with a total score range of 0-56. They have been widely used in both clinical practice and research activities.

# *EEG preprocessing*

# Semi-automated EEG pre-processing for artifact rejection was performed using the EEGLAB toolbox [1] and ICLabel toolbox [2]. The preprocessing steps are briefly described as follows. (1) Resampled the raw data to 250 Hz; (2) Visually inspected the EEG signals and manually removed bad segments contaminated by movement artifacts; (3) Applied bandpass filter of 0.5-100 Hz and notch filter of 50 Hz to the down-sampled signals; (4) Discarded bad channels by checking the spectra of all channels. The rejected bad channels were then interpolated using neighboring channels via spherical spline interpolation [3]; (5) Removed the remaining artifacts using independent component analysis (ICA). In this step, ICs marked by ICLables as eye movement, muscle, heart, channel noise and line noise were rejected, and ICs labelled as other would be checked by the operators manually to decide to keep or reject; (6) EEG data were re-referenced to the common average. (7) The resulting EEG data were finally visually inspected again for any possible remaining noise.

# For frequency analysis, we estimated the power spectral density using Welch’s technique with 50% overlapping Hamming windows of 10-second epochs in length. Spectral densities were derived from the same canonical frequency bands as described in the main body.

# *Source Localization*

# All connectivity analyses were carried out in source space. Source localization was performed using the Brainstorm toolbox [4]. The head model was computed using OpenMEEG with FreeSurfer average brain template and symmetric boundary element method [5]. A total of 3003 rotating dipoles with unconstrained orientations were generated on the cortical surface. After co-registration of electrode locations and anatomical MRI, the lead-field matrix was obtained. The inverse operator that maps current density from sensor space to the source space was then estimated by the minimum norm estimation method (MNE) with depth weight and regularization. For each vertex, the current density time series were reduced to a single principal direction by principal component analysis (PCA). Finally, the analytical data were binned into 20 seconds time epochs.

# *Estimating Functional Connectivity*

# The 31 cortical ROIs (14 bilateral and 3 midline ROIs) were defined by independent parcellation of resting-state fMRI connectivity using ICAs from 38 healthy participants [6]. Here, we chose the debiased weighted phase-lag index (dwPLI) to represent the non-zero phase-lag statistical interdependencies between each pair of ROIs [7]. dwPLI is a modified version of the phase lag index that minimizes the contributions of volume conduction and field spread, which could bias the estimation even at the source level. For each pair of regions, the connectivity was represented by averaging the dwPLI values over all possible vertex pairs. Consequently, 465 regional pairwise connectivity features were identified for each participant.

# Supplementary Figures and Tables

##
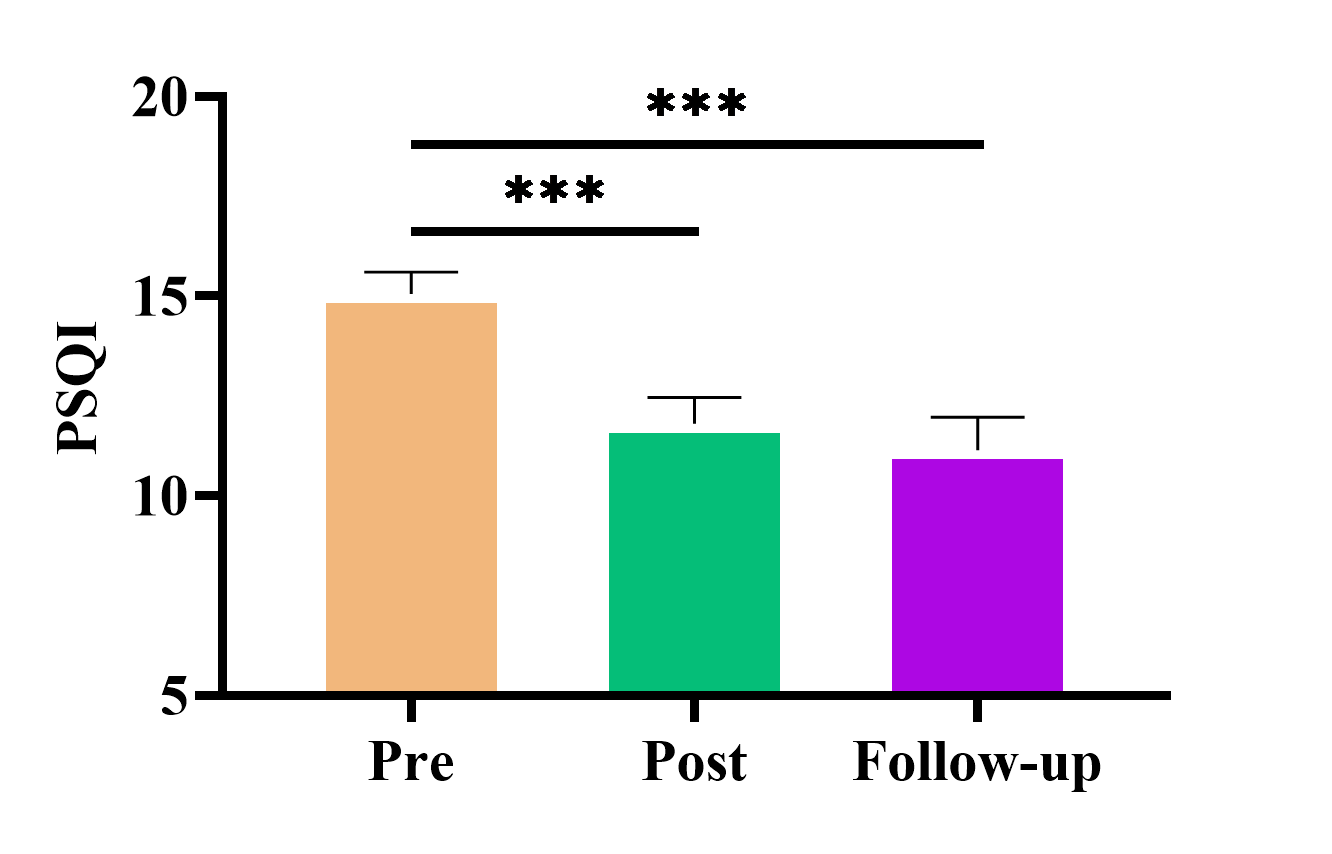


**Figure 1:** Clinical result of PSQI at three time points: pre-treatment, post-treatment and 1-month after rTMS completion. Note: *p<0.05, **p<0.01, ***p<0.001.


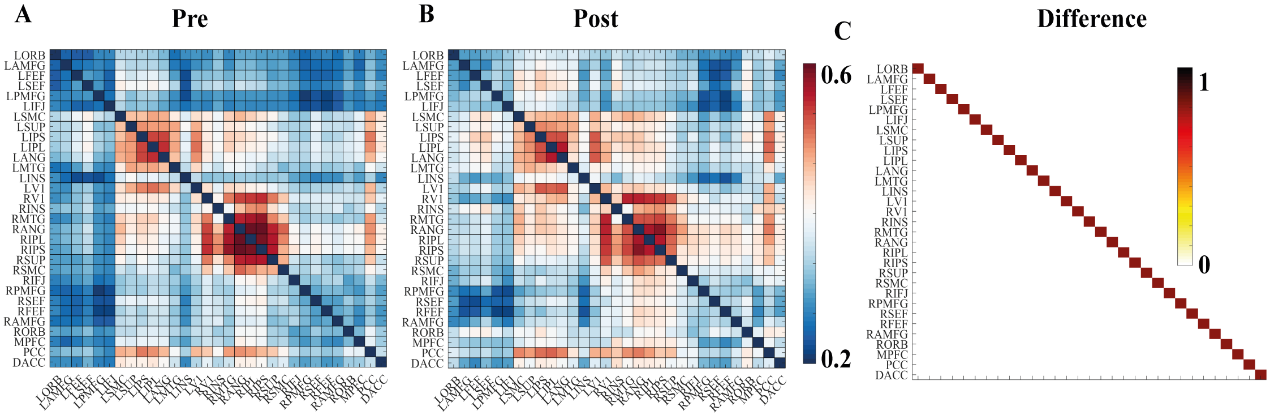


**Figure 2:** Functional connectivity of alpha band (8-13 Hz) before and after rTMS. (A) dwPLI connectivity matrices of the CID group pre-rTMS. (B) dwPLI connectivity matrices of the CID group post-rTMS. (C) The network-based statistics results of post-rTMS > pre-rTMS. There was no significant difference between the pre- and post-matrices survived the NBS correction at p <0.05.

**Table 1:** ROI parcellation [8] :

| Abbreviation | Brain regions |
| --- | --- |
| LV1 | Left Visual Area 1 |
| RV1 | Right Visual Area 1 |
| LSMC | Left Somatosensory Cortex |
| RSMC | Right Somatosensory Cortex |
| LIFJ | Left Inferior Frontal Junction |
| RIFJ | Right Inferior Frontal Junction |
| LIPS | Left Intraparietal Sulcus |
| RIPS | Right Intraparietal Sulcus |
| LFEF | Left Frontal Eye Fields |
| RFEF | Right Frontal Eye Fields |
| LSEF | Left Supplemental Eye Fields |
| RSEF | Right Supplemental Eye Fields |
| PCC | Posterior Cingulate Cortex |
| MPFC | Medial Prefrontal Cortex |
| LANG | Left Angular Gyrus |
| RANG | Right Angular Gyrus |
| LPMFG | Left Posterior Middle Frontal Gyrus |
| RPMFG | Right Posterior Middle Frontal Gyrus |
| LIPL | Left Inferior Parietal Lobule |
| RIPL | Right Inferior Parietal Lobule |
| LORB | Left Orbital Gyrus |
| RORB | Right Orbital Gyrus |
| LMTG | Left Middle Temporal Gyrus |
| RMTG | Right Middle Tempotal Gyrus |
| LAMFG | Left Anterior Middle Frontal Gyrus |
| RAMFG | Right Anterior Middle Frontal Gyrus |
| LINS | Left Insula |
| RINS | Right Insula |
| DACC | Dorsal Anterior Cingulate Cortex |
| LSUP | Left Supramarginal Gyrus |
| RSUP | Right Supramarginal Gyrus |

**Table 2:** 34 pairs among all 31 brain parcellations showed significant differences between the pre and post EEG scans (All pairs survived NBS correction, p <0.01).

| Pair | Brainnetome regions | *Pair* | Brainnetome regions |
| --- | --- | --- | --- |
| 1 | LIFJ - LFEF | 2 | RIFJ - LFEF |
| 3 | LIFJ - LSEF | 4 | RIFJ - LSEF |
| 5 | RSMC-MPFC | 6 | LIFJ-MPFC |
| 7 | LFEF-MPFC | 8 | LIFJ-LPMFG |
| 9 | RIFJ-LPMFG | 10 | MPFC-LPMFG |
| 11 | LFEF-RPMFG | 12 | LSEF-RPMFG |
| 13 | RSEF-RPMFG | 14 | LPMFG-RPMFG |
| 15 | LIFJ-RORB | 16 | LFEF-RORB |
| 17 | LPMFG-RORB | 18 | RIFJ-LAMFG |
| 19 | MPFC-LAMFG | 20 | RORB-LAMFG |
| 21 | LSEF-RAMFG | 22 | LPMFG-RAMFG |
| 23 | RMTG-RAMFG | 24 | LIFJ-LINS |
| 25 | MPFC-LINS | 26 | LPMFG-LINS |
| 27 | MPFC-RINS | 28 | RIFJ-DACC |
| 29 | RPMFG-DACC | 30 | LIFJ-LSUP |
| 31 | LPMFG-LSUP | 32 | MPFC-RSUP |
| 33 | RPMFG-RSUP | 34 | RAMFG-RSUP |

**Table 3:** Multiple linear model of clinical outcomes and connectivity changes

| Variables | Estimate | Std.Error | *t* value | *p* value |
| --- | --- | --- | --- | --- |
| (intercept) | 0.165 | 0.253 | 0.651 | 0.52 |
| Gender | -0.02 | 0.093 | -0.22 | 0.827 |
| Age | -0.005 | 0.004 | -1.283 | 0.209 |
| HAMA | 0.013 | 0.008 | 1.612 | 0.118 |
| HAMD | 0.007 | 0.011 | 0.659 | 0.515 |
| LIFJ-LINS | 0.761 | 0.237 | 3.207 | 0.00326 |
| MPFG-LINS | -0.539 | 0.187 | -2.878 | 0.00745 |

**REFERENCE:**

# [1] Delorme A, Makeig S. EEGLAB: an open source toolbox for analysis of single-trial EEG dynamics including independent component analysis. J Neurosci Methods 2004;134:9–21. https://doi.org/10.1016/j.jneumeth.2003.10.009.

# [2] Pion-Tonachini L, Kreutz-Delgado K, Makeig S. ICLabel: An automated electroencephalographic independent component classifier, dataset, and website. NeuroImage 2019;198:181–97. https://doi.org/10.1016/j.neuroimage.2019.05.026.

# [3] Perrin F, Pernier J, Bertrand O, Echallier JF. Spherical splines for scalp potential and current density mapping. Electroencephalogr Clin Neurophysiol 1989;72:184–7. https://doi.org/10.1016/0013-4694(89)90180-6.

# [4] Tadel F, Baillet S, Mosher JC, Pantazis D, Leahy RM. Brainstorm: a user-friendly application for MEG/EEG analysis. Comput Intell Neurosci 2011;2011:879716. https://doi.org/10.1155/2011/879716.

# [5] Gramfort A, Papadopoulo T, Olivi E, Clerc M. OpenMEEG: opensource software for quasistatic bioelectromagnetics. Biomed Eng Online 2010;9:45. https://doi.org/10.1186/1475-925X-9-45.

# [6] Chen AC, Oathes DJ, Chang C, Bradley T, Zhou Z-W, Williams LM, et al. Causal interactions between fronto-parietal central executive and default-mode networks in humans. Proc Natl Acad Sci U S A 2013;110:19944–9. https://doi.org/10.1073/pnas.1311772110.

# [7] Vinck M, Oostenveld R, van Wingerden M, Battaglia F, Pennartz CMA. An improved index of phase-synchronization for electrophysiological data in the presence of volume-conduction, noise and sample-size bias. NeuroImage 2011;55:1548–65. https://doi.org/10.1016/j.neuroimage.2011.01.055.

# [8] Toll RT, Wu W, Naparstek S, Zhang Y, Narayan M, Patenaude B, et al. An Electroencephalography Connectomic Profile of Posttraumatic Stress Disorder. Am J Psychiatry 2020;177:233–43. https://doi.org/10.1176/appi.ajp.2019.18080911.
